# Solution approach using heuristic and artificial neural networks methods in assembly line balancing problems: A case study in the lighting industry

**DOI:** 10.1016/j.heliyon.2024.e26950

**Published:** 2024-03-01

**Authors:** Yelda Karatepe Mumcu

**Affiliations:** Department of Electric and Energy, University of Marmara, P.O Box 34865, Istanbul, Turkey

**Keywords:** *Heuristic*, A*ssembly line balancing*, *Lighting automation manufacturing*, *Artificial neural networks*

## Abstract

Assembly line efficiency is one of the most important parameters that determine the overall efficiency of a manufacturing company. The production of a product under optimum conditions is ensured by a balanced assembly. With a balanced assembly line, machinery, material and labour costs are reduced. Within the scope of this research, real data about the daily production capacity and assembly line efficiency of a company producing Emergency Luminaire were taken, the same assembly line was balanced with 4 different Heuristic ALB methods and the results were compared. According to the results obtained, a high line efficiency of 93.955% was achieved using the Hoffman, Comsoal and Moodie&Young (M&Y) methods, and 84.414% was achieved with the Ranked Positional Weight (RPW) method. As a result of this, it was observed that the daily production capacity increased from 250 units to 375 units. As a result of the study, it was revealed that the efficiency of the existing assembly line and accordingly the daily production capacity increased. In addition, the study results of this assembly line were taught to an artificial neural network model for training purposes, and the work station results of the operations of a different assembly line were obtained with 99.940 accuracy. In this context, it has been revealed that the artificial neural networks method can be used in addition to the use of the heuristic method in the solution of ALB problems.

## 1 Introduction

Production systems are living systems that are affected by changes and developments occurring worldwide. So much so that these changes and developments directly affect the consumers, and the affected consumer behaviours also affect the companies, in other words, production systems. First, the pandemic, then the war, and the subsequent high inflation had a significant impact on the whole world, therefore on both producers and consumers. The decrease in consumer purchasing power has forced companies to abandon the habits they exhibited during high-profit margin periods and has pushed them to exhibit more rigorous behaviours in terms of efficiency and cost.

There are many ways to increase efficiency, but some of them (modernization, automation and technological improvements) require new investments [1].

At a time when efficiency is so important, balanced assembly lines emerge as one of the keys to achieving a higher level of efficiency with the available facilities without making any investment in terms of production systems. This will eliminate waste, reduce production costs and provide a competitive advantage to companies [2]. While assembly lines, one of the most popular production methods among flow production systems, are widely used in the production of high-quality standard products, they have now become important in the production of small quantities of special products [3].

Assembly lines; forms the basis of many production systems, especially in the automotive, electronics and white goods industries. An assembly line consists of a succession of workstations where workpieces are transferred continuously along the line by means of labour or material handling equipment. At each workstation, tasks, each part of the assembly process, are performed repetitively and thoroughly to produce the final product [4]. The purpose of the system is to assemble the components of a product and obtain the finished product [5].

The most basic problem encountered in assembly lines; is a balanced assignment of tasks to workstations, considering one or more purposes, under some constraints depending on the production system and product. This problem is called the ALB problem [6].

**Fig. 1:**
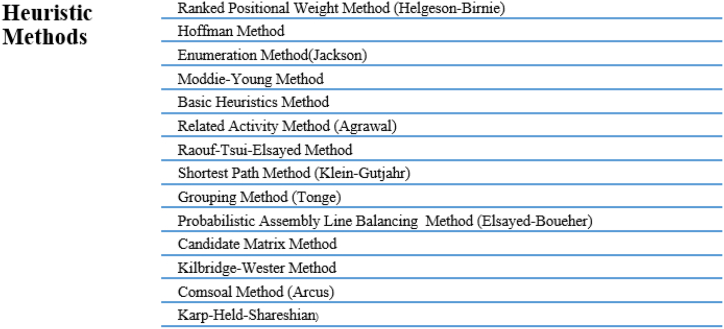
Heuristic methods.

The methods developed to solve this problem are called ALB methods, and they can be basically classified as single-model, multi-model and mixed-model assembly lines according to their production methods [7]. Fig. 1 shows the heuristic methods used. Analytical methods and simulation techniques are also used in ALB solution approaches [8,9].

This study was carried out in order to increase the efficiency of the assembly line in a company that manufactures Emergency Luminaires. In this context, the emergency luminaire production (ELP) process was examined and the data required for ALB were obtained. In parallel with the data obtained, ALB studies were carried out with intuitive methods called Hoffman Method, Comsoal Method, Ranked Positional Weight Method and Moodie & Young Method. In addition, obtained data from ALB studies were taught to an artificial neural network model for training purposes, and the workstation results of the operations of a different assembly line were obtained.

## 2 Literature review

The first ALB research was implemented in the automotive industry. Nowadays, ALB works are carried out in almost every sector.

When examining the history of ALB studies, the idea of ALB was first mentioned by Ref. [10] in the article titled “Continuous Production Line Balancing”. The first research published in this field is the work named “Assembly Line Balancing Problem” published by Ref. [11].

After this study, various studies were carried out by many researchers. Today, these methods are named after the researchers who found the method. They can be listed in chronological order as follows; [[12], [13], [14], [15], [16], [17], [18], [19], [20], [21], [22], [23], [24], [25]].

When balanced assembly line studies are examined in the lighting sector, it is seen that they are few in number. Ling [26] obtained results that would increase the stability and efficiency of the production line. Yao [27], who examined the workstations on the lamp assembly line and their effects on the line production capacity, proposed a new line balancing scheme that eliminated these deficiencies. Saptari, Xin and Mohammad [28] examined the efficiency of an existing electrical accessory producing assembly line in Malaysia in their study and suggested that a new assembly line they proposed would achieve more optimum results. LCD TV assembly line was handled and line balancing was performed using two different approaches, and the results of both approaches were compared using the simulation method by Büyüksaatçi, Tüysüz & Bilen [29] in their study. Tuncel and Topaloğlu [4] conducted an assembly line balancing study in order to increase production efficiency in a company that produces electronic goods.

In order to produce the final product on assembly lines, the general purpose is to group assembly operations and to assign this grouping to work stations in the most optimal way. During this process, the priority relationship between the operations is respected and the total assembly time remains the same [30].

In the researchers examined, various soft computing approaches draw attention in addition to heuristic methods in ALB solutions. While using these methods, there are prominent many factors that should be considered in the optimization of the assembly line.

In the researches where the optimization of the assembly line is optimized in minimum cycle time: Genetic algorithm approach [30,31], Petri net approach [32] and other soft computing [33] approaches were used as methods.

In researches in which the assembly line is optimized by assigning minimum workstations, Art colony optimization approach [34], immune algorithm approach [35] and other soft computing [36,37] approaches are used as methods.

In the researches in which the maximum line efficiency of the assembly line is optimized: Genetic algorithm [38], Petri net approach [39,40] were used. In addition, in researches where the assembly line is optimized with minimum idle time, Art colony optimization approach [41] was used as a method.

The aim of this study is to create a new assembly line using heuristic ALB methods to increase the efficiency of the existing assembly line. Another aim is to demonstrate the applicability of heuristic ALB methods in the lighting industry with using artificial neural network method.

## 3 Material and Methodology

### 3.1 Product information

**Fig. 2:**
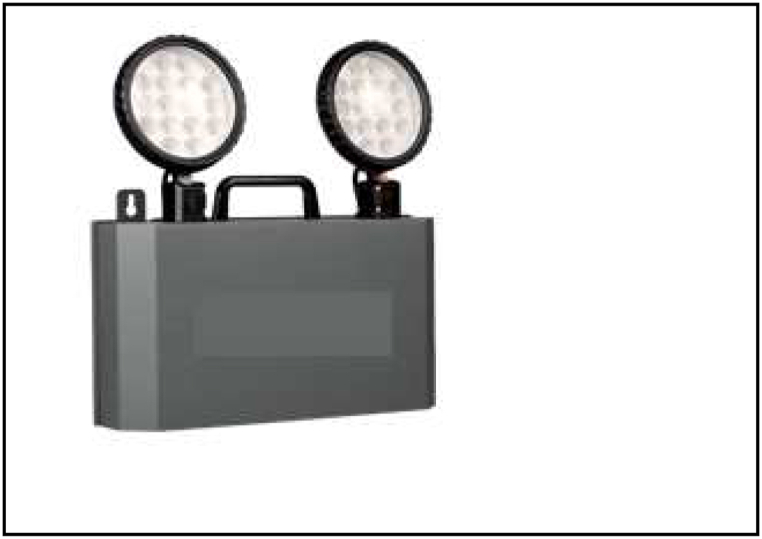
Emergency luminaire (EL).

In this study, the Emergency Luminaire (EL) was examined. Within the scope of the study, the necessary data about the operations, operation times and the machines used for the EL produced by X lighting company were obtained from the managers. The EL, consisting of the body, light source and accumulator, is shown in Fig. 2.

**Fig. 3:**
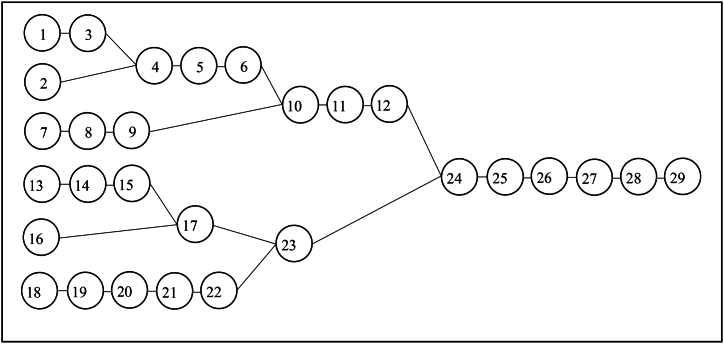
Priority diagram for EL.

**Fig. 4:**
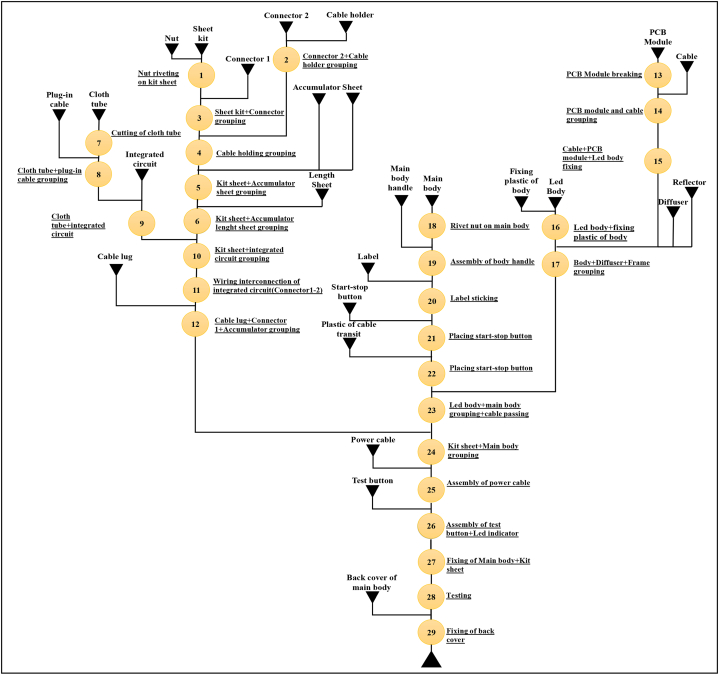
ELP operation flow chart.

The operations, operation times, machines used during the operations and previous operations are seen in the work flow (Fig. 3) and the table (Table 1). In this study, 2 × 5W emergency lighting fixture with portable charging head was chosen to be examined. This product contains 29 operations (Fig. 3). Fig. 4 shows the production flow for EL.

**Table 1:** Operation list for EL production.

Op. No	Operations	Machine Type	Operation Times (min.)	Previous Operations
1	Nut riveting on kit sheet	Nut Riveting	0.280	–
2	Connector 2+Cable holder grouping	Handmade	0.105	–
3	Sheet kit + Connector grouping	E. Screwdriver	0.372	1
4	Cable holding grouping	Pop rivet	0.287	2–3
5	Kit sheet + Accumulator sheet grouping	E. Screwdriver	0.334	4
6	Kit sheet + Accumulator length sheet grouping	E. Screwdriver	0.305	5
7	Cutting of cloth tube	Handmade	0.046	–
8	Cloth tube + plug-in cable grouping	Handmade	0.331	7
9	Cloth tube + integrated circuit	Handmade	0.366	8
10	Kit sheet + integrated circuit grouping	Handmade	1.333	6–9
11	Wiring interconnection of integrated circuit (Connector1-2)	E. Screwdriver	0.800	10
12	Cable lug + Connector 1+Accumulator grouping	E. Screwdriver	0.816	11
13	PCB Module breaking	Handmade	0.098	–
14	PCB module and cable grouping	Handmade	0.396	13
15	Cable + PCB module + Led body fixing	Handmade	0.550	14
16	Led body + fixing plastic of body	E. Screwdriver	0.241	–
17	Body + Diffuser + Frame grouping	Handmade	0.466	15–16
18	Rivet nut on main body	Nut riveting	0.600	–
19	Assembly of body handle	E. Screwdriver	0.376	18
20	Label sticking	Handmade	0.366	19
21	Placing start-stop button	Handmade	0.133	20
22	Placing start-stop button	Handmade	0.138	21
23	Led body + main body grouping + cable passing	Percussive nutrunner	0.743	17–22
24	Kit sheet + Main body grouping	E. Screwdriver	0.700	12–23
25	Assembly of power cable	Handmade	0.733	24
26	Assembly of test button + Led indicator	Handmade	0.678	25
27	Fixing of Main body + Kit sheet	E. Screwdriver	0.600	26
28	Testing	Handmade	0.135	27
29	Fixing of back cover	E. Screwdriver	0.383	28
**Total Time**			**12.684**

### 3.2 Current status in the company

Company X created an assembly line for the production of EL with 10 operators with a total production time of 12.684 min (Table 1) and determined its daily production capacity to be 425 units. When the company records are examined, it is seen that the daily production amount for the last 5 days has been realized as 250 units on average and in parallel with this the efficiency of the assembly line has been realized as 58.82%. In parallel with these data, the cycle time was calculated as 1.27 min for the daily total production amount target of 425 units and as 2.16 min for the actual daily total production amount.

The formulations and calculations on which the data of entity X are based are given in Eqs. (1), (2), (3), (4), (5), (6), (7) [42,43].

(1) $$DTPA=\left[\frac{\;NO\;x\;DTPT}{ST}\right]$$

Where;

*DTPA* is Daily Total Production Amount, *NO* is Number of Operators, *DTPT* is Daily Total Production Time and *ST* is the standard time.

(2) $$DTPA=\frac{\left(10\;x\;540\right)}{12.684}=425\;pieces/day$$

(3) $$Efficiency\;\%=\frac{Output}{Input}\;x\;100$$

(4) $$E\%=\frac{250}{425}\;x\;100=58.82\%$$

(5) $$CT=\left[\frac{\;DTPT}{DTPA}\right]$$

Where;

CT is Cycle Time

(6) $$CT=\left[\frac{\;540}{425}\right]=1.27\;\text{minute}\;\left(\text{for}\;\text{the}\;\text{daily}\;\text{total}\;\text{production}\;\text{amount}\;\text{target}\right)$$

(7) $$CT=\left[\frac{\;540}{250}\right]=2.16\;\text{minute}\;\left(\text{for}\;\text{the}\;\text{actual}\;\text{daily}\;\text{total}\;\text{production}\;\text{amount}\right)$$

### 3.3 Preliminary calculations for re-assembly line balancing

When the EL production operation times are examined, it is seen that the “Kit sheet + integrated circuit grouping” operation has the highest operation time (1.33 min). Since the duration of a workstation cannot be shorter than the longest duration of a work unit and cannot be longer than the cycle time [1], the cycle time can be taken as a minimum of 1.33 min or more. Therefore, in this study, the cycle time was accepted as 1.35 min and in parallel, the total daily production amount was determined as 400 in Eqs. (8), (9).

(8) $$DTPA=\left[\frac{\;DTPT}{CT}\right]$$

(9) $$DTPA=\frac{540}{1.35}=400\;pieces/day$$

Using the formulas shown in Eqs. (10), (11)), loss of balance, efficiency value and daily total production amount of the assembly line are calculated.

(10) $$LB=\left[\frac{\left(nC-\sum C_{0}\right)}{nC}\right]\times 100$$

(11) LE=(1−LB)×100

Where;

*LB* is Loss of Balance (%), *LE* is Line Efficiency (%), *n* is Total Number of Work Stations (number-unit), *C*_*o*_ is Average of Work Station Time (minute) [44].

In this study, it is assumed that handwork operations are performed by all operators with the same type of machines.

### 3.4 Application of heuristic ALB methods

#### 3.4.1 Hoffman Method

According to the Hoffman Method, assignment is made as follows:

- First of all, a matrix is figure out. Since the number of operations for the product examined in this study is 29, the matrix to be prepared is created as 31 × 30 (row x column).
- For operations with a priority relationship, 1 is written in the intersection cells of the relevant operations. Other cells are left blank.
- The sum of the values written in the cells is written in the code row at the bottom.
- The assignment process starts from the first column with the value “0” in the code row.
- The first operation with a value of “0” is assigned. The remaining time of the workstation is found by subtracting the processing time of the relevant operation from the cycle time.
- If there is no other operation with a value of “0”, a new matrix is figure out by deleting the row and column of the relevant operation in the matrix.
- If there is another operation with a value of “0” and the duration of this operation is less than the remaining time of the workstation and the operation is performed on the same type of machine, that operation is also assigned to the workstation. In this case, the rows and columns of the 2 operations with the value “0” are deleted and a new matrix is figure out.

The priority matrix of the product examined within the scope of this study is shown in Table 2–a. As can be seen from the table, the number of operations with a value of “0” is 6 (1,2, 7, 13, 16 and 18).

**Table 2:** Solution matrix.

Op.	1	2	3	4	5	6	7	8	9	10	11	12	13	14	15	16	17	18	19	20	21	22	23	24	25	26	27	28	29	Op	3	4	5	6	8	9	10	11	12	14	15	16	17	19	20	21	22	23	24	25	26	27	28	29
**1**			**1**																											**3**		**1**																						
**2**				**1**																										**4**			**1**																					
**3**				**1**																										**5**				**1**																				
**4**					**1**																									**6**							**1**																	
**5**						**1**																								**8**						**1**																		
**6**										**1**																				**9**							**1**																	
**7**								**1**																						**10**								**1**																
**8**									**1**																					**11**									**1**															
**9**										**1**																				**12**																			**1**					
**10**											**1**																			**14**											**1**													
**11**												**1**																		**15**													**1**											
**12**																								**1**						**16**													**1**											
**13**														**1**																**17**																		**1**						
**14**															**1**															**19**															**1**									
**15**																	**1**													**20**																**1**								
**16**																	**1**													**21**																	**1**							
**17**																							**1**							**22**																		**1**						
**18**																			**1**											**23**																			**1**					
**19**																				**1**										**24**																				**1**				
**20**																					**1**									**25**																					**1**			
**21**																						**1**								**26**																						**1**		
**22**																							**1**							**27**																							**1**	
**23**																								**1**						**28**																								**1**
**24**																									**1**					**29**																								
**25**																										**1**				**Code No**	**0**	**1**	**1**	**1**	**0**	**1**	**2**	**1**	**1**	**0**	**1**	**0**	**2**	**0**	**1**	**1**	**1**	**2**	**2**	**1**	**1**	**1**	**1**	**1**
**26**																											**1**																											
**27**																												**1**																										
**28**																													**1**																									
**29**																																																						
**Code** **No**	**0**	**0**	**1**	**2**	**1**	**1**	**0**	**1**	**1**	**2**	**1**	**1**	**0**	**1**	**1**	**0**	**2**	**0**	**1**	**1**	**1**	**1**	**2**	**2**	**1**	**1**	**1**	**1**	**1**																									

First, operation number 1 is assigned to 1st workstation. Since the cycle time is 1.35 min and the processing time of the 1st operation is 0.280 min, the remaining time of the 1st workstation is calculated as 1.350–0.280 = 1.053 min.

Then, it is checked whether the 2nd operation can be performed on the 1st workstation (machine type). Since the 2nd operation is a handmade operation and the processing time is less than the remaining time of the 1st workstation, it is assigned to the 1st workstation. The remaining time of the 1st workstation is found as 1.053–0.105 = 0.965 min.

In the same way, operations 7, 13 and 18 are assigned and the remaining time of the 1st workstation is calculated as 0.965–0.046 = 0.919 min, 0.919–0.098 = 0.821 min, 0.821–0.600 = 0.221 min respectively.

Here, operation no. 16 is not assigned to 1st workstation. Because the relevant operation is performed with a different type of machine.

After the assignment of 5 operations with values “0” (1,2, 7, 13 and 18) is performed, a new matrix is figure out by deleting rows and columns 1,2,7,13 and 18 in the priority matrix (Table 2–b).

All operations are assigned to workstations according to the assignment systematic described above (Table 3).

**Table 3:** Line balancing results.

Workstation Number	Operation No	Machine Type	Time (min.)	Total Time for Workstation (x)	Remaining Time (C-x)
1	1	Nut Riveting	0.280	1.262	0.071
	2	Handmade	0.105		
	7	Handmade	0.046		
	13	Handmade	0.098		
	18	Nut Riveting	0.600		
	21	Handmade	0.133		
2	3	E. screwdriver	0.372	1.34	0.010
	8	Handmade	0.331		
	14	Handmade	0.396		
	16	E. screwdriver	0.241		
3	4	Pop rivet	0.287	1.341	0.009
	9	Handmade	0.366		
	15	Handmade	0.550		
	22	Handmade	0.138		
4	5	E. screwdriver	0.334	1.256	0.094
	17	Handmade	0.466		
	19	E. screwdriver	0.376		
	11	E. screwdriver	0.080		
5	6	E. screwdriver	0.305	1.349	0.001
	20	Handmade	0.366		
	26	Handmade	0.678		
6	10	Handmade	1.333	1.333	0.017
7	12	E. screwdriver	0.816	1.334	0.016
	28	Handmade	0.135		
	29	E. screwdriver	0.383		
8	23	Percussive nutrunner	0.743	0.743	0.607
9	24	E. screwdriver	0.700	1.300	0.050
	27	E. screwdriver	0.600		
10	25	Handmade	0.733	0.733	0.617
	***Total Time***		***12.684***	***12.684***	***1.509***

The assembly line was created with 10 workstations and a cycle time of 1.35 min, and the balance loss and line efficiency were calculated with the formulas given in Eqs. (12), (13)).

(12) $$LB=\left[\frac{\left[\left(10\times 1.350\right)-\left(12.684\right)\right]}{\left(10\times 1.350\right)}\right]\times 100=6.044\%$$

(13) LE=(1−0.060)×100=93.955%

#### 3.4.2 COMSOAL method

The assignment is made according to the following steps:

Step 1 First of all, the following table needs to be constituted (Table 4–a). There are 3 columns in this table. The first column indicates the operation numbers, the second column indicates the amount of previous operations (APO) the relevant operation, and the third column indicates the operations without any previous operations (OWPO) before.

**Table 4:** Solution stages of problem using of COMSOAL method.

Op. No	APO	OWPO	Op. No	APO	OWPO	Op. No	APO	OWPO
1	**0**	1	3	**0**	3	4	**0**	4
2	**0**	2	4	1	8	5	1	9
3	1	7	5	1	14	6	1	15
4	2	13	6	1	16	9	**0**	19
5	1	16	8	**0**	18	10	2	
6	1	18	9	1		11	1	
7	**0**		10	2		12	1	
8	1		11	1		15	**0**	
9	1		12	1		17	1	
10	2		14	**0**		19	**0**	
11	1		15	1		20	1	
12	1		16	**0**		21	1	
13	**0**		17	2		22	1	
14	1		18	**0**		23	2	
15	1		19	1		24	2	
16	**0**		20	1		25	1	
17	2		21	1		26	1	
18	**0**		22	1		27	1	
19	1		23	2		28	1	
20	1		24	2		29	1	
21	1		25	1				
22	1		26	1				
23	2		27	1				
24	2		28	1				
25	1		29	1				
26	1							
27	1							
28	1							
29	1

Step 2 Assigning operations starts with the operations in the 3rd column and the first operation is assigned to the 1st workstation. Other operations are respectively assigned to the same workstation, considering the operation time, workstation remaining time and machine type. After the assignment process, the assigned operations are deleted from the table, APO and OWPO columns are recreated and this assignment systematic is applied to all operations.

In Table 4, all steps regarding the application of the method are given as in the example.

First of all, operation number 1, which is in the first row in the 3rd column, is assigned to 1st workstation and the workstation remaining time is calculates (1.35–0.280 = 1.070 min).

The times of the operations numbered 2, 3 and 13 in Table 4–a which in 3rd column is shorter than the residual time of 1st work station. They can be assigned to 1st work station. The type of machines which is used during the operation is different the type of the machine in the first work station though its operation time is 0.241 min. Therefore, it cannot be assigned to 1st work station.

Afterwards the operations numbered 1, 2, 7 and 13 are deleted from Table 4–a and Table 4–b is derived.

This assignment system continues by taking the same rules into account for other operations and all operations are assigned to workstations.

The solution results according to designing assembly line by using COMSOAL Method are shown Table 5 (see Table 6).

**Table 5:** Line balancing results.

Workstation Number	Operation No	Machine Type	Time (min.)	Total Time for Workstation (x)	Remaining Time (C-x)
1	1	Nut Riveting	0.280	1.262	0.071
	2	Handmade	0.105		
	7	Handmade	0.046		
	13	Handmade	0.098		
	18	Nut riveting	0.600		
	21	Handmade	0.133		
2	3	E. screwdriver	0.372	1.340	0.010
	8	Handmade	0.331		
	14	Handmade	0.396		
	16	E. screwdriver	0.241		
3	4	PPM	0.287	1.341	0.009
	9	Handmade	0.366		
	15	Handmade	0.550		
	22	Handmade	0.138		
4	5	E. screwdriver	0.334	1.256	0.094
	17	Handmade	0.466		
	19	E. screwdriver	0.376		
	11	E. screwdriver	0.080		
5	6	E. screwdriver	0.305	1.349	0.001
	20	Handmade	0.366		
	26	Handmade	0.678		
6	10	Handmade	1.333	1.333	0.017
7	12	E. screwdriver	0.816	1.334	0.016
	28	Handmade	0.135		
	29	E. screwdriver	0.383		
8	23	Pop rivet	0.743	0.743	0.607
9	24	E. screwdriver	0.700	1.300	0.050
	27	E. screwdriver	0.600		
10	25	Handmade	0.733	0.733	0.617
	***Total Time***		***12.684***	***12.684***	***1.509***

**Table 6:** Solution of problem using by RPW Method.

Op. No	**Time (min.)**	1	2	3	4	5	6	7	8	9	10	11	12	13	14	15	16	17	18	19	20	21	22	23	24	25	26	27	28	29	Ranked Positional Weight Value
1	0.280			1	+	+	+				+	+	+												+	+	+	+	+	+	7.036
2	0.105				1	+	+				+	+	+												+	+	+	+	+	+	6.489
3	0.372				1	+	+				+	+	+												+	+	+	+	+	+	6.756
4	0.287					1	+				+	+	+												+	+	+	+	+	+	6.384
5	0.334						1				+	+	+												+	+	+	+	+	+	6.097
6	0.305										1	+	+												+	+	+	+	+	+	5.763
7	0.046								1	+	+	+	+												+	+	+	+	+	+	6.201
8	0.331									1	+	+	+												+	+	+	+	+	+	6.155
9	0.366										1	+	+												+	+	+	+	+	+	5.824
10	1.333											1	+												+	+	+	+	+	+	5.458
11	0.080												1												+	+	+	+	+	+	4.125
12	0.816																								1	+	+	+	+	+	4.045
13	0.098														1	+		+						+	+	+	+	+	+	+	5.482
14	0.396															1		+						+	+	+	+	+	+	+	5.384
15	0.550																	1						+	+	+	+	+	+	+	4.988
16	0.241																	1						+	+	+	+	+	+	+	4.679
17	0.466																							1	+	+	+	+	+	+	4.438
18	0.600																			1	+	+	+	+	+	+	+	+	+	+	5.585
19	0.376																				1	+	+	+	+	+	+	+	+	+	4.985
20	0.366																					1	+	+	+	+	+	+	+	+	4.609
21	0.133																						1	+	+	+	+	+	+	+	4.243
22	0.138																							1	+	+	+	+	+	+	4.110
23	0.743																								1	+	+	+	+	+	3.972
24	0.700																									1	+	+	+	+	3.229
25	0.733																										1	+	+	+	2.529
26	0.678																											1	+	+	1.796
27	0.600																												1	+	1.118
28	0.135																													1	0.518
29	0.383																														0.383

The assembly line was created with 10 workstations and a cycle time of 1.35 min, and the balance loss and line efficiency were calculated with the formulas given in Eqs. (14), (15)).

(14) $$LB=\left[\left[\frac{\left(10\times 1.35\right)-\left(12.684\right)}{\left(10\times 1.35\right)}\right]\right]\times 100=6.044\%$$

(15) LE=(1−0.060)×100=93.955%

#### 3.4.3 Ranked Positional Weight Method (RPW)

The assignment is done according the following steps:

• Priority diagram is created.
• The position weight value is calculated for each task. The position weight of a task is equal to the sum of the duration of that task and the duration of the tasks that are successors to that task.
• Tasks are sorted from largest to smallest according to position weight.
• Tasks are assigned to stations in order, giving priority to the highest position weight.
• If the station time exceeds the cycle time when the next task is assigned, the next task is attempted to be assigned as long as it does not violate priority relations. If there are no tasks available for assignment, a new station opens.
• The last two steps are repeated until all tasks are assigned to stations.

The remaining time of 1st work station is calculated as 1.35–0.280 = 1.070 min. After the assignment of operation numbered 1, the time of the operation numbered 2 which has higher position weight and it can be assignment to 1st work station. The remaining time of the 1st work station is calculated as 1.070–0.105 = 0.965 min.

After the assignment of operation numbered 2, although the time of the operation numbered 3 which has higher position weight is shorter than remaining time of this operation, because of different type of machines are used, it cannot be assigned.

The operation numbered 3 is assigned to 2nd work station. The residual time of 2nd work station is 1.35–0.372 = 0.978 min.

The rest of the operations are assignment according to precedence and type of the machines. The solution results of the RPW method used for assembly line balancing of the EL product are shown in Table 7.

**Table 7:** Line balancing results.

Workstation Number	Operation No	Machine Type	Time (min.)	Total Time for Workstation (x)	Remaining Time (C-x)
1	1	Nut riveting	0.280	1.226	0.124
	2	Handmade	0.105		
	7	Handmade	0.046		
	8	Handmade	0.331		
	9	Handmade	0.366		
	13	Handmade	0.098		
2	3	E. screwdriver	0.372	1.252	0.098
	5	E. screwdriver	0.334		
	6	E. screwdriver	0.305		
	16	E. screwdriver	0.241		
3	4	Pop rivet	0.287	1.233	0.117
	14	Handmade	0.396		
	15	Handmade	0.550		
4	18	Nut riveting	0.600	1.237	0.133
	20	Handmade	0.366		
	21	Handmade	0.133		
	22	Handmade	0.138		
5	10	Handmade	1.333	1.333	0.017
6	19	E. screwdriver	0.376	1.057	0.293
	17	Handmade	0.466		
	11	E. screwdriver	0.080		
	28	Handmade	0.135		
7	12	E. screwdriver	0.816	1.199	0.151
	29	E. screwdriver	0.383		
8	23	Percussive nutrunner	0.743	0.743	0.607
9	24	E. screwdriver	0.700	1.300	0.050
	27	E. screwdriver	0.600		
10	25	Handmade	0.733	0.733	0.617
11	26	Handmade	0.678	0.678	0.672
	***Total Time***		***12.684***	***12.684***	***2.879***

The assembly line was created with 11 workstations and a cycle time of 1.35 min, and the balance loss and line efficiency were calculated with the formulas given in Eqs. (16), (17)).

(16) $$LB=\left[\left[\frac{\left(11\times 1.35\right)-\left(12.684\right)}{\left(11\times 1.35\right)}\right]\right]\times 100=14.585\%$$

(17) LE=(1−0.145)×100=85.414%

#### 3.4.4 Moddie & Young Method (M&Y)

The assignment is done according the following steps:

• In the table consisting of rows with the operation numbers: the first column shows the previous operation number(s), the second column shows the next operation number(s). The last column shows processing time (Table 8).

**Table 8:** Solution stages of problem using of M & Y method.

Op. No	Previous Operation(s)	Next Operation(s)	**Time (min.)**	Control	Op. No	Previous Operation(s)	Next Operation(s)	**Time (min.)**	Control
1	–	3	0.280	–	3	–	4	0.372	–
2	–	4	0.105	–	4	3	5	0.287	
3	1	4	0.372		5	4	6	0.334	
4	2–3	5	0.287		6	5	10	0.305	
5	4	6	0.334		8	–	9	0.331	–
6	5	10	0.305		9	8	10	0.366	
7	–	8	0.046	–	10	6–9	11	1.333	
8	7	9	0.331		11	10	12	0.080	
9	8	10	0.366		12	11	24	0.816	
10	6–9	11	1.333		14	–	15	0.396	–
11	10	12	0.080		15	14	17	0.550	
12	11	24	0.816		16	–	17	0.241	–
13	–	14	0.098	–	17	15–16	23	0.466	
14	13	15	0.396		19	–	20	0.376	–
15	14	17	0.550		20	19	21	0.366	
16	–	17	0.241	–	21	20	22	0.133	
17	15–16	23	0.466		22	21	23	0.138	
18	–	19	0.600	–	23	17–22	24	0.743	
19	18	20	0.376		24	12–23	25	0.700	
20	19	21	0.366		25	24	26	0.733	
21	20	22	0.133		26	25	27	0.678	
22	21	23	0.138		27	26	28	0.600	
23	17–22	24	0.743		28	27	29	0.135	
24	12–23	25	0.700		29	28	–	0.383	
25	24	26	0.733						
26	25	27	0.678						
27	26	28	0.600						
28	27	29	0.135						
29	28	–	0.383
• For operations that are not preceded by an operation, a "-" sign is placed in the first column [45].

Since the operation with the longest duration is the 18th operation, it is assigned to 1st workstation. The remaining time from 1st workstation is found as 1.350–0.600 = 0.750 min. Among the remaining operations, operation 1 has the longest duration. Since it is suitable in terms of time and machine type, it can be assigned to 1st workstation (0.750–0.280 = 0.470-min remaining time). Operations 2, 13 and 7, which meet the same conditions from the control column in Table 8–a, are assigned to the 1st workstation.

The assigned operations are deleted and Table 8–b is obtained. The same process is applied for the operations in Table 8–b and assignments to workstations are made considering time and machine type.

All operations are assigned to workstations according to the assignment systematic described above (Table 9).

**Table 9:** Line balancing results.

Workstation Number	Operation No	Machine Type	Time (min.)	Total Time for Workstation (x)	Remaining Time (C-x)
1	18	Nut riveting	0.600	1.262	0.088
	1	Nut riveting	0.280		
	2	Handmade	0.105		
	13	Handmade	0.098		
	7	Handmade	0.046		
	21	Handmade	0.133		
2	14	Handmade	0.396	1.282	0.068
	19	E. Screwing	0.376		
	3	E. screwdriver	0.372		
	22	Handmade	0.138		
3	15	Handmade	0.550	1.233	0.117
	20	Handmade	0.366		
	8	Handmade	0.331		
	11	E. screwdriver	0.080		
4	9	Handmade	0.366	1.208	0.142
	16	E. screwdriver	0.241		
	17	Handmade	0.466		
	28	Handmade	0.135		
5	23	Percussive nutrunner	0.743	0,743	0.607
6	4	Pop rivet	0.287	1.020	0.330
	25	Handmade	0.733		
7	5	E. screwdriver	0.334	1.339	0.011
	6	E. screwdriver	0.305		
	24	E. screwdriver	0.700		
8	10	Handmade	1.333	1.333	0.017
9	12	E. screwdriver	0.816	1.199	0.151
	29	E. screwdriver	0.383		
10	26	Handmade	0.678	1.278	0.072
	27	E. screwdriver	0.600		
	***Total Time***		***12.684***	***12.684***	***1.509***

The assembly line was created with 10 workstations and a cycle time of 1.35 min, and the balance loss and line efficiency were calculated with the formulas given in Eqs. (18), (19)).

(18) $$LB=\left[\frac{\left(10\times 1.35\right)-\left(12.684\right)}{\left(10\times 1.35\right)}\right]\times 100=6.044\%$$

(19) LE=(1−0.060)×100=93.955%

### 3.5 Application of artificial neural network method (ANN)

As seen in many multidisciplinary studies that have done by the researchers [[46], [47], [48], [49], [54], [53], [52], [51], [50]], artificial neural networks have shown that they can be applied to any problem as a method that can solve complex problems inspired by biological nervous system easily, quickly and with scientific accuracy. The artificial neural network was trained with the data set of the study, the time of the operations of the production of emergency fixtures with 29 operations, the machine types, the priority order of the operations, the weight distributions of the operations and the efficiency values of each station. Table 10 shows the data of the trained neural network model.

**Table 10:** ANN model training data of EL.

OpERATION No.	mACHINE TYPE	**OpERATION time (min)**	Relationship between opeRATION		Heuristic Methods	WORK station Efficiency	Line Efficiency	WORK Station
			Previous	After				
1	1-Nut riveting 2-Handmade 3-E.Screwdriver 4-Pop rivet 5- Percussive nutrunner	0.280	0	2	1-Hoffman 2-Comsoal 3- RPW 4-M&Y	94.740	93.955	1
.		.	.	.		.	.	.
.		.	.	.		.	.	.
.		.	.	.		.	.	.
.		.	.	.		.	.	.
29		0.383	28	0		98.814	93.955	7

**Fig. 5:**
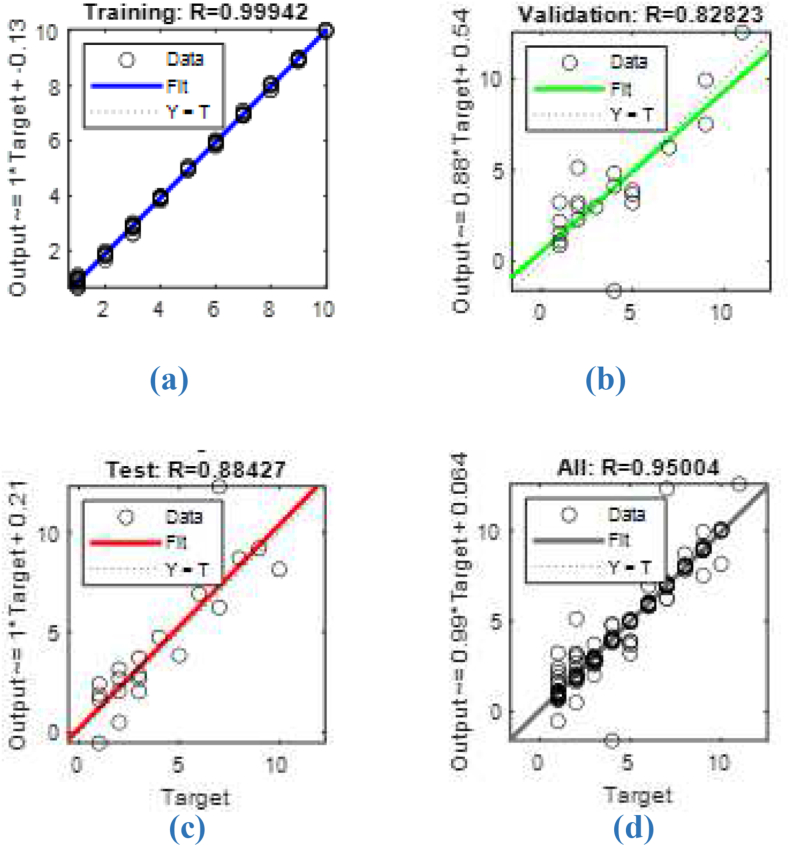
Training regression of ANN model.(a) Training results, (b) Validation results, (c) Test results, (d) All results.

**Fig. 6:**
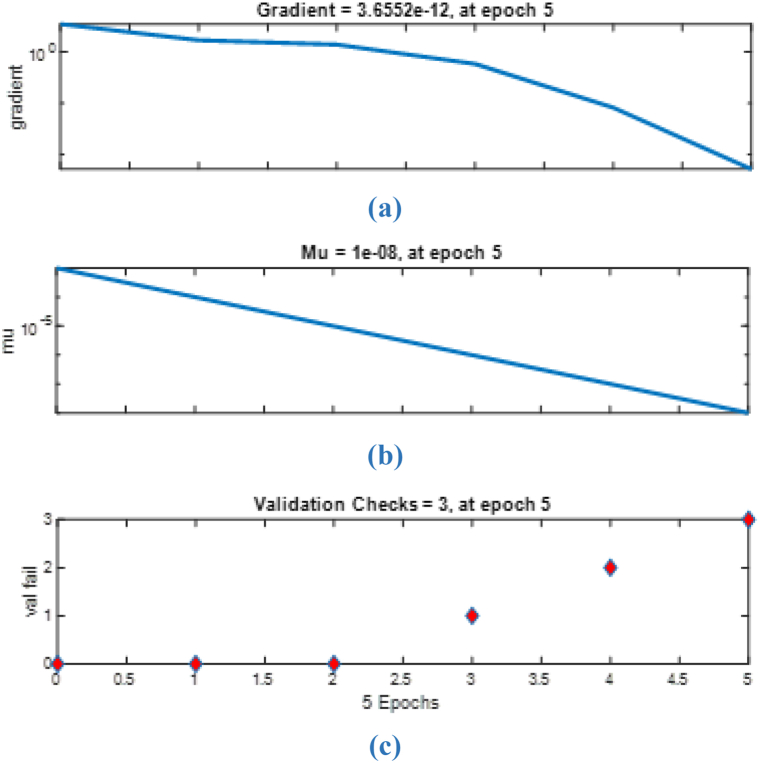
Training state plot of ANN model (a) Gradient value, (b) Mu value, (c) Validation checks.

After determining the most suitable set of input variables for the neural network model, the Levenberg-Marquart algorithm was chosen for learning purposes. By using the data input set of the artificial neural network model, numbered of the work station assigned to the operations according to the heuristic method type constitute the output data set. 70% of the data will be used to train the network and 15% will be used for testing. The remaining 15% is reserved for verification. As can be seen from the data set results used for training purposes in Fig. 5, Fig. 6, the learning was performed with 99.9% accuracy.

## 4 Results

The results of the assembly line work carried out for EL production line balancing are given in Table 11.

**Table 11:** Results of studies for ALB using four Heuristic method.

Work-station	Assembly Line Balancing Methods							
	Hoffman		Comsoal		RPW		M & Y	
	Op.	Eff. (%)	Op.	Eff. (%)	Op.	Eff. (%)	Op.	Eff. (%)
1	1	93.481	1	93.481	1	93.407	18	93.481
	2		2		2		1	
	7		7		7		2	
	13		13		8		13	
	18		18		9		7	
	21		21		13		21	
2	3	99.259	3	99.259	3	92.740	14	94.962
	8		8		5		19	
	14		14		6		3	
	16		16		16		22	
3	4	99.333	4	99.333	4	91.333	15	98.296
	9		9		14		20	
	15		15		15		8	
	22		22				11	
4	5	93.037	5	93.037	18	91.629	9	89.481
	17		17		20		16	
	19		19		21		17	
	11		11		22		28	
5	6	99.925	6	99.925	10	98.740	23	55.037
	20		20					
	26		26					
6	10	98.740	10	98.740	19	78.296	4	75.555
					17			
					11		25	
					28			
7	12	98.814	12	98.814	12	88.814	5	99.185
	28		28		29		6	
	29		29				24	
8	23	55.037	23	55.037	23	55.037	10	98.740
9	24	96.296	24	96.296	24	96.296	12	88.814
	27		27		27		29	
10	25	54.296	25	54.296	25	54.296	26	94.666
							27	
11	–	–	–	–	26	50.222	–	–
Line Eff. (%)	**93.955**		**93.955**		**84.414**		**93.955**	
Loss of Balance (%)	**6.044**		**6.044**		**14.585**		**6.044**	

Line efficiency is the ratio of the total processing time at all workstations multiplied by the cycle time and the number of workstations. The efficiency of the line is calculated as in Eq. (20) with heuristic methods used for ALB solution in EL production.

(20) $$LE=\frac{\sum_{i=1}^{m}{Pt}_{i}}{m\times C_{t}}\times 100$$

With *m* is number of work station, *P*_*t*_ is processing time in work station *i*_th_ and *C*_*t*_ is cycle time.

**Fig. 7:**
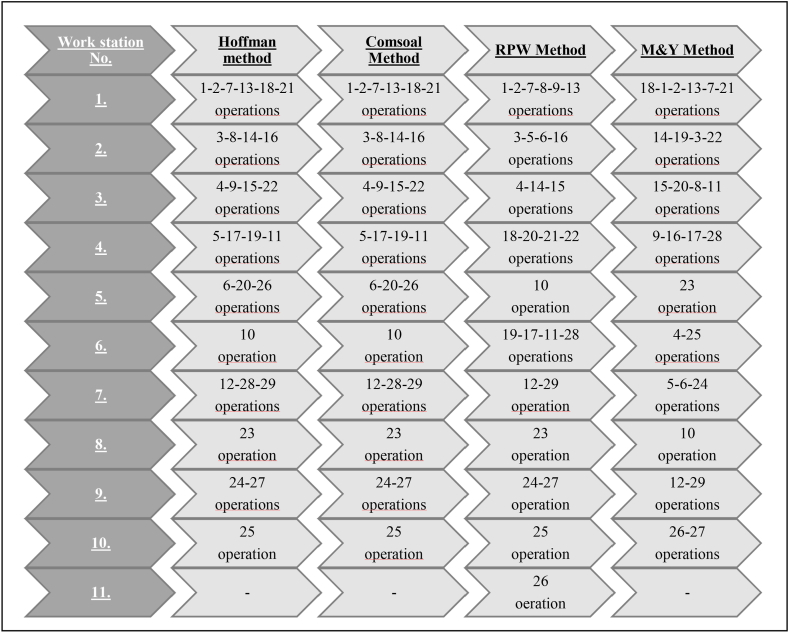
Line results according to four heuristic methods.

Loss of balance and assembly line efficiency of designed assembly line is shown Fig. 7 for four heuristic methods.

As can be seen from the table, while the line was balanced with 10 workstations in the ALB study performed with the Hoffman, Comsoal and M&Y Methods, line balancing was performed with 11 workstations in the RPW Method. In summary, when the methods used in ALB are examined in terms of line efficiency, high line efficiency has been achieved with all methods.

However, considering the current production line conditions, the necessity of 11 workstations in line balancing performed with the RPW method requires the use of an additional operator. In that case, it can be stated that the line balancing performed with this method is not suitable for the operation. When the results of the other three methods were examined, the efficiency of the existing production line was 58.82%, while the line efficiency was 93.955%. This shows that an increase of 35.130% was achieved in assembly line efficiency. When these results are examined in terms of daily total production capacity, 250 Emergency Lighting Fixtures are produced per day with the existing assembly line, while it will be possible to produce 375 units per day with the newly created assembly lines by Hoffman, Comsoal and Moodie&Young methods.

Again, as can be seen in Table 11, when the operation assignments to the workstations are examined, it is seen that the results of the Hoffman method and the Comsoal method are exactly the same. The reason for the difference in the operation assignments made to the workstations is that the systematics of the methods are different.

The result of the heuristic methods used in assembly lines are shown Fig. 7 and the results of the network model created by the artificial neural network method are shown Table 12.

**Table 12:** Results of ANN model studies for ALB.

Algorithm		Levenberg-Marquardt		
Data division		Random		
Performance		Mean squared error		
		Observations	MSE	R
Training Set	Training	96	0.0145	99.94%
	Validation	20	3.1689	82.82%
	Test	20	2.3202	88.43%
Test Set	Test	26	18.1971	79.52%

Work stations, which were determined according to 4 different heuristic methods recommended to be used in the production of EL, were applied to the ANN model for training purposes. After the training of the generated ANN model, learning was provided with an accuracy of 99.940% regression, 20 of the input set were used for validation and the other 20 for testing, and the training average was determined as 95.004%.

In order to test the usability of the generated ANN model in solving a different product ALB problem, the operation steps of a lighting luminaire produced in the same company were applied to the model as a test set. The test set was prepared considering the criteria used in the input data set.

**Fig. 8:**
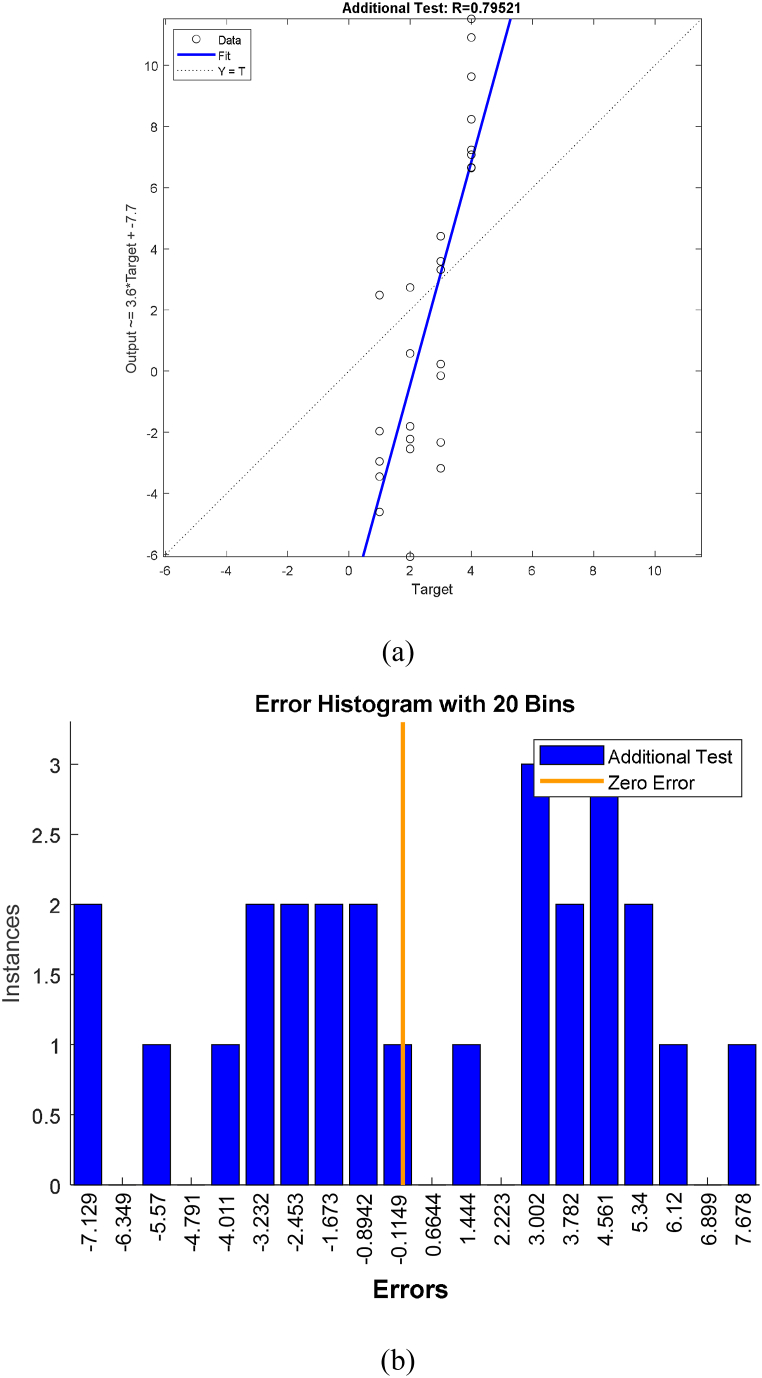
(a) ANN testing model regression results (b) ANN testing model error results.

The estimation of the operations that should be assigned to the work stations belonging to the lighting fixture ALB problem used for test purposes gave test results with an accuracy of 79.521%. It has been demonstrated that in the solution of ALB problems, artificial neural network method can be used as well as heuristic methods (Fig. 8).

## 5 Conclusion

The aim of this study is to examine the applicability of intuitive ALB methods to design the highest efficiency assembly lines in lighting automation companies.

Within the scope of the study, a line balancing study was carried out in a company that produces ELs by using 4 heuristic.

The efficiency of the existing production line and the total daily production have been increased significantly. This leads to the conclusion that higher efficiency can be produced under the same conditions. From this point of view, productivity is the biggest weapon in the hands of businesses. Of course, productivity can be increased in enterprises with investments based on economic power, but using existing opportunities without wasting will increase operating efficiency without the need for any investment. One of the best examples and application areas of this is assembly lines.

An increase of 125 units in the daily production amount of an enterprise with an average daily production amount of 250 units also shows that the current production can be increased by 50% with the same opportunities. This means reaching production targets in a shorter time with less cost (labour, energy, etc.). This study also revealed that all companies operating in the electricity sector can achieve high line efficiency by using these methods in the production of different products.

In addition to these methods, it has been demonstrated that artificial neural networks can be used for the formation of assembly lines and be assignment of operations to work stations. It will shed light on the applicability of the results, especially for researchers and manufacturers, in terms of further studies on this subject and the application of other Heuristic Methods and machine learning methods.

This study can be further expanded by using other assembly line balancing methods in assembly line balancing studies on the production of different products produced in this sector (complex products with more operations and using different machines). Although three methods gave the same results in this study, different results may be obtained in studies involving different products. In the ANN part, it may be possible to further improve the results by using different algorithms.

## Funding

None.

## Data availability statement

All data generated or analysed during this study are included in article.

## Additional information

No additional information is available for this paper.

## CRediT authorship contribution statement

**Yelda Karatepe Mumcu:** Writing – review & editing, Writing – original draft, Methodology.

## Declaration of competing interest

The authors declare that they have no known competing financial interests or personal relationships that could have appeared to influence the work reported in this paper.
